# Longitudinal drop-out and weighting against its bias 

**DOI:** 10.1186/s12874-017-0446-x

**Published:** 2017-12-08

**Authors:** Steffen C. E. Schmidt, Alexander Woll

**Affiliations:** Institute of Sport and Sport Science, Karlsruhe Institute of Technology, 76131 Karlsruhe, Germany

**Keywords:** Representativeness, Weighting, Drop-out, Logistic regression, MoMo

## Abstract

**Background:**

The bias caused by drop-out is an important factor in large population-based epidemiological studies. Many studies account for it by weighting their longitudinal data, but to date there is no detailed final approach for how to conduct these weights.

**Methods:**

In this study we describe the observed longitudinal bias and a three-step longitudinal weighting approach used for the longitudinal data in the MoMo baseline (*N* = 4528, 4–17 years) and wave 1 study with 2807 (62%) participants between 2003 and 2012.

**Results:**

The most meaningful drop-out predictors were socioeconomic status of the household, socioeconomic characteristics of the mother and daily TV usage. Weighting reduced the bias between the longitudinal participants and the baseline sample, and also increased variance by 5% to 35% with a final weighting efficiency of 41.67%.

**Conclusions:**

We conclude that a weighting procedure is important to reduce longitudinal bias in health-oriented epidemiological studies and suggest identifying the most influencing variables in the first step, then use logistic regression modeling to calculate the inverse of the probability of participation in the second step, and finally trim and standardize the weights in the third step.

## Background

Representativeness with reference to the target population is a crucial factor when conducting large population-based epidemiological studies [1–4]. In order to obtain correct estimates for prevalence and means, a high participation rate is considered necessary [5]. However, even high participation rates do not exclude potential sample bias due to intercorrelation between nonresponse and characteristics of interest [3]. Many studies have shown that the health profile of non-participants in epidemiological studies is worse compared to participants [6–8]. Each study has its individual design and subsequent problems but there is broad agreement among methodologists that a weighting procedure including a weighting for selection bias, an adjustment factor for potential responder bias, trimming to control additional variance and poststratification to define the target population [3, 4, 9] improves representativeness in cross-sectional studies. In sample survey methodology, these weights are often estimated as the inverse of the probability of selection [10, 11].

The problem gets even more difficult when a longitudinal approach is used. When an initial sample is considered representative of the target population, drop-out bias in subsequent waves can result in losing representativeness of the sample if unit nonresponse is assumed to occur not at random (NMAR) [12]. Decreasing rates of participation are a major concern in longitudinal population-based studies and have been reported from nearly every large study center [1, 3]. For a detailed description of longitudinal data bias with different types of drop-out characteristics see Mazumdar and colleagues [13]. Data from follow-up investigations is also used to uncover cohort effects and treated as if it was gathered from true cross-sectional studies [1]. However, this leads to unpredictive errors from no to very high bias in outcomes dependent on the characteristics of interest [1].

Therefore, weighting according to the probability of participation in order to control for potential responder bias on target variables is suggested for longitudinal studies. This probability can be estimated using either weighting classes or logistic regression modeling and is called inverse probability weighting (IPW) [14]. However, one should know that weighting procedures result in an unwanted increase in the estimator’s variance, which can be expressed as

$$ {V}_w={V}_{\mu}\left(1+{s}_w^2\right) $$

where V_w_ represents the weighted estimate’s variance, V_μ_ the unweighted and s^2^ the variance of the weights, assumed to be scaled to average unity [3, 15]. To lower the amount of additional variance, the weights are often modified using trimming [16], collapsing weight strata [17, 18] or shrinking [19, 20]. The amount of increase in variance is often expressed as the efficiency of a weighting procedure, with higher efficiency standing for a lower increase in variance after weighting.

Although IPW is well defined, not every longitudinal study uses and/ or reports a weighting procedure. In our research, we found a lack of comparative data from weighting procedures of longitudinal studies that focus on being representative for a target population. The Motorik-Modul (MoMo) Study is a typical example for a longitudinal, health-oriented study. We gathered nationwide data in Germany, weighted the baseline participants for selection and responder bias and then followed this representative sample in a longitudinal design. In this paper, we describe the effect of drop-out on the central parameters measured in MoMo from baseline to wave 1 and how we compensated for those effects by weighting to create an unbiased longitudinal sample of German children and adolescents.

## Methods

### MoMo design and sample characteristics

The MoMo study [21] is a module of the German Health Interview and Examination Survey for Children and Adolescents (KiGGS) conducted by the Robert Koch Institute (RKI). The aim of the MoMo study is, on the one hand, to gather nationwide representative data on physical fitness, physical activity (PA) and health parameters of children and adolescents, and on the other hand, to create knowledge about the development and interaction of former parameters over time.

To ensure a diverse sample of children and adolescents with primary residence in Germany, the RKI and the German Centre for Surveys, Methods and Analysis (GESIS, formerly ZUMA) drew a nationwide stratified multi-stage probability sample with three evaluation levels at KiGGS baseline [22–24]. First, a systematic sample of 167 primary sampling units was selected from an inventory of German communities stratified according to the BIK classification system that measures the level of urbanization and geographic distribution [24]. Second, an age-stratified sample of randomly selected children and adolescents, with a total of 17,641 participants aged 0–17 years, was drawn from the official registers of local residents [23]. Third, 7866 children and adolescents aged 4–17 years were randomly assigned to the MoMo baseline sample. Of these 7866 children and adolescents, 4529 (57.6%) ultimately participated in the MoMo baseline study from 2003 to 2006. Out of those, a total of 2807 (62.0%) children and adolescents participated in MoMo wave 1. The data collection of wave 1 began in September 2009 and ended in July 2012.

### Research methods

During the KiGGS survey [24], a wide range of data on the health of German children and adolescents were collected. Apart from interviews, the study also included physical examinations (including laboratory analyses of blood and urine samples) and tests. The thematic focus of the KiGGS baseline study was on health status, health behavior, living conditions, protection and risk factors and utilization of services provided by the health system.

The MoMo longitudinal study included a physical fitness test profile as well as a PA questionnaire [25] and anthropometric measurements. The physical fitness test profile included endurance (cardiorespiratory fitness), strength (upper and lower limbs), gross motor coordination (dynamic and static balance), fine motor coordination (manual dexterity, reaction time) and flexibility [26]. All participants completed a standardized PA questionnaire to declare overall PA (in the past 7 days and in a normal week), everyday PA (duration, frequency, type), sports activity at school as well as in and outside organized clubs (duration, frequency, intensity, type, seasonality) and PA related questions like family support and overall sport interest.

### Statistical Methods & Modelling

One of the main objectives of the MoMo study is to describe the development of motor performance, PA and anthropometrics of children during adolescents. In order to avoid underestimation of potential negative developments due to NMAR unit nonresponse of unfit and/or overweight children, the longitudinal sample was weighted in a three-step IPW procedure. IPW is recommended in literature for dealing with NMAR unit nonresponse in longitudinal designs [27, 28]. The three-step weighting procedure results in a longitudinal weight for each longitudinal participant.

### Step 1: Variable selection and preparing the data

Since one goal of the MoMo study was to create representative^1^ data for Germany, an initial weight was applied for every baseline participant. MoMo is a subsample of the representative KiGGS survey and did not draw participants directly from the residents’ registration office. Therefore the initial weighting procedure is not typical for cross-sectional studies and will not be described in detail in this paper. In a nutshell, we conducted a design weighting which adjusted the MoMo subsample to the KiGGS sample, followed by trimming and post stratification using the data from the German Micro Census 2004 in order to reflect the distributions of sex, age, region, migration status and education in Germany. The KiGGS weighting procedure is described elsewhere [23]. These initial baseline weights were used during all latter steps.

To select potentially significant covariates for predicting wave 1 participation, a nonresponse analysis (weighted by baseline weights) was conducted. Because we had access to both, KiGGS and MoMo responses, we had a large amount of health, PA, fitness, anthropometric and social background-related data. In a preliminary screening, 979 variables that were not measured in every participant were excluded. A total of 1152 remaining items and relevant indices measured at baseline were then combined in 33 context themes. Some examples for different themes are activity pattern, anthropometrics, motor performance, social background and status, family characteristics and climate, psychological peculiarity and residential neighborhood.

Categorical variables were made from metrical ones by using practical implication whenever possible (for example BMI), or dividing into five percentile-based groups if no practical implication was available (for example: media use, birth weight). Missing values among covariates could not be assumed to be missing completely at random (MCAR) or missing at random (MAR), as, for example, questions about the household’s income are selectively missing in families with very low and high income. Hence an extra category for missing values was defined for each covariate. This technique of dealing with NMAR missing data in covariates is suggested when the aim of the regression model is creating predictors and the reason for missing data is the person refusing to answer [29]. When potential drop-out predictors from different previous measurement points are used and there are various reasons for non-monotone missing data, using multiple imputation to deal with missing data in covariates is suggested [30].

For each context theme, a multivariate regression model with least absolute shrinkage and selection operator (LASSO) and 10-fold cross-validation was conducted to rank variables according to their power of predicting drop-out. LASSO is based on shrinkage estimation, and handles the multicollinearity problem [31]. Although LASSO is not yet very common in epidemiological research [32], it has been welcomed in the literature for variable selection [33], especially in logistic regression with high numbers of covariates [34]. Lasso shrinks unstable estimates to zero and excludes variables without the need for formal statistical testing [32]. Among the 33 context themes, LASSO revealed 116 significant predictors for drop-out. To further reduce the number of potential predictors, we merged the significant predictors into six larger context themes and ran LASSO regression models again to rank variables according to their power of predicting drop-out. To determine a practicable number of predictors for the longitudinal model in step 2, we used a bootstrapping method that has been shown to be a viable method in reducing unnecessary complexity and overfitting [31]. We started with a model containing only the three top-ranked predictors in each theme and calculated the area under the receiver operating characteristic curve (AUC). We then continued increasing the number of predictors by rank, and stopped when the AUC did not increase significantly (*p* > .05). This reduced the total number of potential predictors to 29.

#### Step 2: The longitudinal model against drop-out bias

After most potential predictors were identified in step 1, we included them in a final multivariate regression model (weighted with baseline weights) using LASSO and a 10-fold cross-validation to obtain the optimal drop-out predictive factor subset.

#### Step 3: Trimming and standardization

The predicted values from the final logistic model (probability of participation) were gathered using the SAVE PRED command in SPSS. The inverse of the probability of participation was calculated according to 1/PRED. The results were then multiplied with the MoMo baseline weights. This is a common strategy to combine two weighting procedures used, for example in the European Community Household Panel [35, 36].

The resulting weights were trimmed at the 0.5 and 99.5 percentiles. Finally, dividing the weights by their mean re-established the original sample size of 2807 longitudinal participants.

## Results

### Drop-out rates

7866 children and adolescents of the KiGGS sample were assigned to the MoMo Baseline Study. From those, 4529 (57.6%) ultimately participated [21]. One participant was excluded from the data set, because he applied to delete his data. From the remaining 4528 baseline participants, 2807 (62.0%) participated in wave 1. The 1721 children and adolescents who dropped out include two cases of death and 25 cases of moving to other countries.

### Final logistic model

The variable selection in step 1 revealed 29 potential drop-out predictors. From those, 19 predictive factors were selected by LASSO in step 2. The weighting efficiency in step 3 was 41.67% and weights range from 0.07 to 8.17. Table 1 shows the selected predictors and their odds ratio (OR) in predicting response.

**Table 1:** Predictors and their OR for wave 1 participation in the step 2 final logistic model

Variable	Value	Odds Ratio	p-value
Age	4	1.00	
	5	1.07 (0.76–1.50)	.71
	6	1.50 (1.06–2.11)	.02
	7	1.14(0.83–1.71)	.42
	8	1.59 (1.13–2.24)	.01
	9	0.98 (0.70–1.38)	.93
	10	0.93 (0.67–1.30)	.69
	11	0.63 (0.46–0.87)	<.01
	12	0.69 (0.50–0.95)	.02
	13	0.56 (0.40–0.77)	<.01
	14	0.50 (0.37–0.69)	<.01
	15	0.50 (0.37–0.69)	<.01
	16	0.61 (0.45–0.83)	<.01
	17	0.72 (0.53–0.99)	.04
Sex	Female	1.00	
	Male	0.84 (0.75–0.95)	<.01
Socioeconomic status (SES)	Percentile 81–100 (high)	1.00	
	Percentile 61–80	0.86 (0.70–1.05)	.13
	Percentile 41–60	0.62 (0.51–0.75)	<.01
	Percentile 21–40	0.47 (0.39–0.57)	<.01
	Percentile 1–20 (low)	0.22 (0.18–0.27)	<.01
	Missing	0.11 (0.03–0.44)	<.01
Household income	Percentile 81–100 (high)	1.00	
	Percentile 61–80	0.90 (0.73–1.10)	.28
	Percentile 41–60	0.72 (0.59–0.88)	<.01
	Percentile 21–40	0.52 (0.43–0.63)	<.01
	Percentile 1–20 (low)	0.31 (0.26–0.37)	<.01
	Missing	0.49 (0.24–1.01)	.05
Mother’s Education (CASMIN)	High (3b, 3a)	1.00	
	Moderate (2a, 2c_gen, 2c_voc)	0.71 (0.58–0.88)	<.01
	Low (1a, 1b, 1c, 2b)	0.33 (0.26–0.40)	<.01
	Missing	0.34 (0.19–0.61)	<.01
Body Mass Index (BMI)	Underweight	1.00	
	Normal weight	1.02 (0.83–1.27)	.85
	Overweight	0.89 (0.69–1.16)	.39
	Obese	0.39 (0.28–0.55)	<.01
	Missing	0.98 (0.33–2.96)	.97
Who does the child live with?	Natural Parents	1.00	
	Mother (with partner)	0.52 (0.45–0.61)	<.01
	Father (with partner)	0.22 (0.12–0.40)	<.01
	Grandparents	0.71 (0.15–3.46)	.67
	Adoptive Parents	0.30 (0.12–0.74)	<.01
	Children’s Home	0.13 (0.01–1.14)	.07
	Missing	1.03 (0.46–2.29)	.94
Different language at home?	No	1.00	
	Yes	0.39 (0.33–0.46)	<.01
	Missing	0.71 (0.36–1.43)	.34
Type of school	Primary	1.00	
	Lower Secondary	0.28 (0.23–0.35)	<.01
	Secondary	0.59 (0.50–0.70)	<.01
	Gymnasium	0.87 (0.74–1.03)	.11
	Comprehensive	0.43 (0.31–0.58)	<.01
	Special	0.18 (0.10–0.33)	<.01
	Missing	0.68 (0.41–1.12)	.68
Has the child repeated a grade?	No	1.00	
	Yes/Missing	0.32 (0.26–0.39)	<.01
Smoking at home	No	1.00	
	Yes	0.51 (0.45–0.58)	<.01
	Missing	0.26 (0.17–0.41)	<.01
Hyperactivity Disorder (ADHS)	No	1.00	
	Unknown	0.82 (0.50–1.35)	.44
	Yes	0.53 (0.42–0.67)	<.01
	Missing	0.18 (0.13–0.25)	<.01
SDQ (Strength and Difficulties Questionnaire)	Percentile 81–100 (few SD)	1.00	
	Percentile 61–80	0.77 (0.63–0.93)	<.01
	Percentile 41–60	0.54 (0.45–0.65)	<.01
	Percentile 21–40	0.52 (0.44–0.62)	<.01
	Percentile 1–20 (many SD)	0.47 (0.39–0.56)	<.01
	Missing	0.14 (0.08–0.26)	<.01
Daily TV usage	0 h	1.00	
	<1 h	0.63 (0.43–0.94)	.02
	1.5 h	0.34 (0.23–0.50)	<.01
	3–4 h	0.10 (0.06–0.16)	<.01
	5 h+	0.02 (0.01–0.10)	<.01
	Missing	0.22 (0.15–0.32)	<.01
Do your parents take you on outings (for example to the cinema)?	Yes, all the time	1.00	
	Sometimes	0.88 (0.75–1.04)	.13
	Rarely	0.54 (0.45–0.64)	<.01
	No	0.47 (0.37–0.61)	<.01
	Missing	0.16 (0.11–0.22)	<.01
Does the child wear a helmet when biking?	Yes	1.00	
	No	0.45 (0.39–0.51)	<.01
	No bike	0.34 (0.26–0.46)	<.01
	Missing	0.21 (0.14–0.31)	<.01
Does the child use toothpaste with fluoride?	Yes	1.00	
	No	1.67 (1.10–2.54)	.02
	Missing	0.45 (0.40–0.51)	<.01
Model information: N: 4528; adjusted R^2^ = 0.25 (Nagelkerke); −2 log Likelihood: 5239.86; AUC: 73.2%			

Regarding personal characteristics, age, sex and BMI were significant predictors of participation in wave 1. The chance of participating decreased with higher age and in children with obesity. However, the OR from overweight children did not differ from normal weight children. The chance of participating  was also lower in boys than in girls. Regarding the children’s activity pattern, only daily TV usage remained in the final model.

The modelling showed that the children’s parents, especially the mother, heavily influenced participation. SES and household income both turned out to be meaningful predictors of participation. The mother’s education, smoking at home and whether the parents often take their children on outings were also selected. Further, whether the child lives at home or elsewhere remained in the final model. The OR for participation of children not living at home was significantly lower, except for children living with their grandparents.

**Fig. 1:**
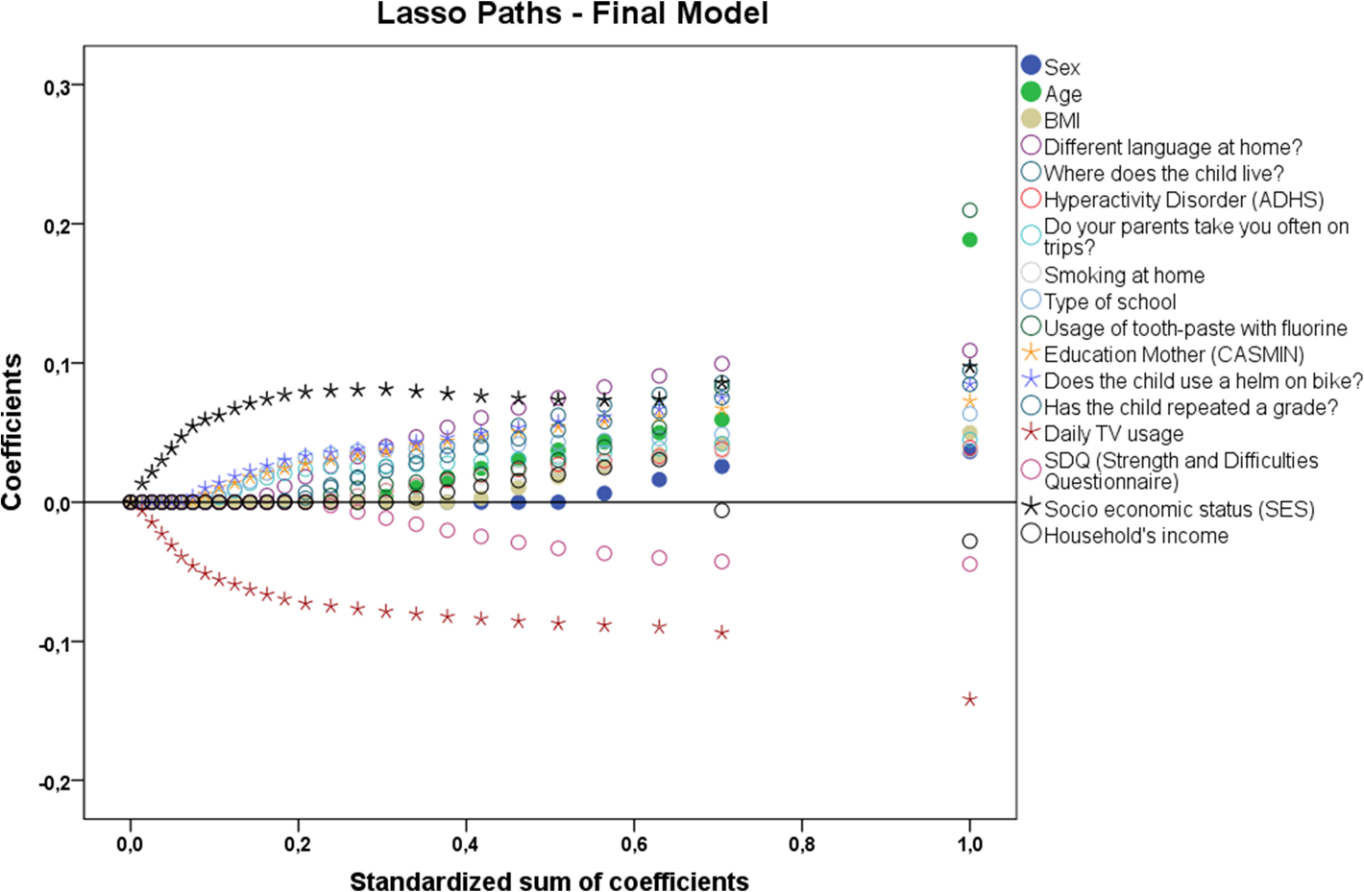
Least absolute shrinkage and selection operator (LASSO) shrinking path diagram

Among migration background variables, whether a different language is spoken at home turned out to be the most meaningful predictor. Other migration variables were eliminated during the LASSO selection. Finally, some rather unexpected variables, including whether the child wears a helmet when biking and whether the child uses toothpaste with fluoride, were selected. Among many health-related variables, including chronic diseases, subjective well-being and blood and urine tests, only hyperactivity disorder was selected in the final model. No motor performance variable was selected. Figure 1 shows the LASSO shrinking paths of the final model.

The LASSO shrinking paths show the order in which less important predictors shrank to zero in the final model. SES, daily TV usage, wearing a helmet when biking and the education of the mother turned out to be the most important drop-out predictors.

### Drop-out bias before and after weighting

The differences in selected baseline variables between wave 1 respondents and nonrespondents are shown in Table 2. The results show that children and adolescents from families with lower SES, as well as children and adolescents with migration backgrounds were less likely to participate in wave 1. General interest in sports showed only a small difference between respondents and nonrespondents. Respondents and nonrespondents also showed no meaningful difference in days per week with 60 min of moderate to vigorous activity, but respondents reported being members of sport clubs more often. Starting at the age of 6, respondents had a 0.5–0.9 point higher BMI and slightly better motor performance compared to nonrespondents.

**Table 2:** Differences between nonrespondents and weighted/unweighted respondents for selected MoMo baseline variables

All participants					
Variable		Whole sample (baseline weight)	Wave 1 nonresp. (baseline weight)	Wave 1 respondents (baseline weight)	Wave 1 respondents (longitudinal weight)
Sex [%]	Male	51.3%	53.6%	46.4%	51.2%
	Female	48.7%	49.5%	50.5%	48.8%
Age [years]		11.3 ± 4.1	12.0 ± 4.0	10.8 ± 4.1	11.3 ± 4.1
Socioeconomic status (Winkler index)	Low	33.7%	46.1%	25.1%	32.9%
	Med	43.9%	39.6%	48.3%	44.8%
	High	20.9%	14.3%	26.6%	21.6%
Mig. Background		16.6%	23.1%	11.4%	15.6%
Sport interest (1: very low, 5: very high)		3.81 ± 1.09	3.76 ± 1.13	3.86 ± 1.05	3.85 ± 1.06
4–5 years					
BMI		15.7 ± 1.8	15.7 ± 2.0	15.7 ± 1.6	15.8 ± 1.8
Standing long jump [cm]		88.6 ± 20.2	85.7 ± 20.1	90.4 ± 20.1	89.9 ± 19.6
Days per week with a minimum of 60 min MVPA		4.9 ± 2.1	4.8 ± 2.2	4.9 ± 1.9	4.9 ± 2.0
Sport club membership		46.8%	32.2%	55.6%	49.5%
Balancing backwards		12.7 ± 8.5	11.3 ± 8.2	13.5 ± 8.6	13.1 ± 8.5
Jumping side to side		10.1 ± 3.8	9.5 ± 3.7	10.4 ± 3.8	10.4 ± 3.8
6–10 years					
BMI		17.0 ± 2.7	17.3 ± 3.0	16.8 ± 2.5	17.0 ± 2.8
Standing long jump [cm]		124.9 ± 21.2	123.8 ± 22.0	125.6 ± 20.7	125.2 ± 20.7
Days per week with a minimum of 60 min MVPA		4.4 ± 2.0	4.3 ± 2.1	4.4 ± 2.0	4.4 ± 2.0
Sport club membership		58.7%	49.1%	63.8%	59.6%
Balancing backwards		27.3 ± 10.1	27.1 ± 10.5	27.4 ± 9.9	27.3 ± 9.8
Jumping side to side		20.6 ± 6.7	20.6 ± 6.7	20.5 ± 6.7	20.5 ± 6.7
11–13 years					
BMI		19.7 ± 4.0	20.2 ± 4.6	19.3 ± 3.2	19.6 ± 3.5
Standing long jump [cm]		153.2 ± 24.0	150.6 ± 24.4	155.9 ± 23.3	154.5 ± 23.3
Days per week with a minimum of 60 min MVPA		3.6 ± 1.9	3.6 ± 2.0	3.7 ± 1.8	3.7 ± 1.9
Sport club membership		56.9%	47.0%	66.8%	62.1%
Balancing backwards		33.0 ± 9.4	31.4 ± 9.7	34.6 ± 8.8	34.0 ± 9.1
Jumping side to side		32.0 ± 6.1	31.4 ± 6.7	32.5 ± 5.5	32.4 ± 5.7
14–17 years					
BMI		21.9 ± 4.0	22.1 ± 4.1	21.6 ± 3.8	21.9 ± 4.5
Standing long jump [cm]		173.4 ± 34.8	172.2 ± 35.6	174.7 ± 33.8	173.6 ± 34.4
Days per week with a minimum of 60 min MVPA		3.3 ± 1.8	3.3 ± 1.9	3.3 ± 1.8	3.3 ± 1.8
Sport club membership		48.8%	45.8%	52.0%	46.6%
Balancing backwards		34.2 ± 9.2	33.8 ± 9.2	34.7 ± 9.2	34.4 ± 9.3
Jumping side to side		35.2 ± 6.5	34.8 ± 6.6	35.7 ± 6.4	35.3 ± 6.8

The longitudinal weights introduced by the weighting procedure reduced the bias between respondents (Table 2 column four) and the whole baseline sample (column one) in a meaningful way, especially for SES and migration background.

## Discussion

### General findings

The comparison between respondents and nonrespondents showed that socioeconomic characteristics had the most striking impact on re-entering the study in wave 1. Among health parameters, only hyperactivity disorder and obesity stayed in the final model. Physical activity and motor performance turned out not to be very meaningful predictors and nonrespondents only differed slightly from respondents. Since our study focuses on fitness, PA and health, we expected those differences to be higher.

The applied longitudinal weights were able to reduce the drop-out bias, even in variables that were not explicitly used in the final logistic regression model (for example the motor performance variables). We assume that the reason for this is intercorrelation between the variables in the logistic model and those that were eliminated in the process. We therefore conclude that the more variables are taken into account during the logistic regression, the better the final result, even for unobserved characteristics of the sample. Studies that focus on being representative of a target population should therefore include a wide range of information about their participants, even if the research question is rather narrowly defined.

However, in large epidemiological studies it is often impossible to include every single variable, interaction or index that has been observed in the logistic modelling. Searching for those variables that are related most closely to the response propensity in a preliminary screening is a common approach in complex data sets [11, 36]. We used a method in which we build context themes among observed variables and then used LASSO to identify the most meaningful predictors in every context theme. This turned out to be a practical method to reduce complexity.

However, we were not able to completely eliminate the drop-out bias in every variable of interest. The reason for this is that longitudinal weights need to be estimated on the basis of available information about the nonrespondents, which is, as opposed to exactly known design weights, an approximation [37].

In general, the extent to which nonresponse or drop-out effects the variables of interest depends on two components: the proportion of non-participants or drop-outs and the degree of systematic differences between participants and non-participants [1]. In a study with many different outcome measures like this one, these systematic differences can differ widely among target variables, making it difficult to decide whether the disadvantages of a complex weighting procedure are a necessary evil or not. A common disadvantage of weighting is an increase in the variance of the estimator [14] and in line with this an increase in the standard errors in conducted analyses [11]. We trimmed the weights at the 0.5 and 99.5 percentiles to reduce this variance [16] and observed an increase in estimator variance (unweighted vs. longitudinal weights) between 5% (age) and 35% (BMI) and a final weighting efficiency of 41.7%. At 41.7%, our final weighting efficiency is only modest but acceptable. However, since especially children and adolescents with extreme values for BMI drop out, we do think that the variance of our unweighted sample underestimates the population’s variance and that the increase in the weighted sample is, at least to some extent, contextually correct. An experimental trimming at the 1.0 and 99.0 percentiles resulted in an increase in efficiency of 2.3%, but at the same time differences between weighted longitudinal respondents and baseline as shown in Table 2 increased in a meaningful way.

### Limitations

The main challenge in applying ideal longitudinal weights is the selection of variables and indices for the logistic model. In our approach, the selection of variables in context themes was not fully objective. For applying cross-sectional weights, which is not described in this paper, we compared our context-theme technique with a fully objective stepwise backwards technique including all variables, and encountered a substantial loss in efficiency of the weights. Another limitation of longitudinal weighting is that the final weights are carried out for the whole sample. Whenever subsamples like males and females or age groups are built in later analyses, weighting those will result in an incorrect number of degrees of freedom in the analyses, and as a consequence thereof, wrong *p*-values and confidence intervals. Theoretically, a new weighting procedure or at least standardization of the weights must be conducted for each subsample in order to avoid this. However this is not always doable in small subsamples and the results would lack comparability. To avoid misleading results, we strongly recommend a comparison between weighted and unweighted statistics in every analysis, even when only weighted data is interpreted.

Another limitation that must be stated is the fact that the method described in this study is only valid if covariates from only one previous measurement point are used. In many longitudinal studies, data from more than one measurement point can be used to predict the participation in later points. In these cases, missing data among covariates shows more complex patterns with persons participating in different measurement points, and literature suggests using multiple imputation to deal with missings in covariates instead of defining an extra category [38].

Lastly, we used information of baseline covariates measured six years before the drop-out or the participation in wave 1 occurred. Among baseline covariates, time-dependent confounders may have changed over time, and especially those variables which are very unstable over time will be underestimated in their power of predicting a drop-out at a later point in time because they have changed and incorrect information is used. To account for this, a nonrespondent telephone interview can be used to gather up-to-date information. However this method is only practical in small samples where the effort is acceptable.

## Conclusion

To date, there is no detailed approach for how to conduct weights in longitudinal studies available. Every study is unique and comes with its unique difficulties, like too many or too few observed variables, missing data or multiple target populations. The technique we describe in this paper turned out to be a practical way to reduce drop-out bias in complex longitudinal data sets with two measurement points. However, whether weighting improves the quality of answers or not is highly dependent on the research question and the study structure. It is good practice to report both weighted and unweighted estimates [38], or at least weighted and unweighted statistics, to provide satisfying information to the reader.

## Abbreviations

BMI: Body Mass Index
GESIS: German Centre for Surveys, Methods and Analysis
KiGGS: German Health Interview and Examination Survey for Children and Adolescents
MoMo: Motorik-Modul
OR: Odds Ratio
PA: Physical Activity
RKI: Robert Koch Institute
SES: Social and economic status

## Footnotes

1 Representative for German children and adolescents in the year of 2004 with respect to sex, age, region, migration status and education
